# Importance of “Process Evaluation” in Audiological Rehabilitation: Examples from Studies on Hearing Impairment

**DOI:** 10.1155/2014/168684

**Published:** 2014-09-03

**Authors:** Vinaya Manchaiah, Berth Danermark, Jerker Rönnberg, Thomas Lunner

**Affiliations:** ^1^Department of Vision and Hearing Sciences, Anglia Ruskin University, Cambridge CB1 1PT, UK; ^2^Linnaeus Centre HEAD, The Swedish Institute for Disability Research, Department of Behavioral Science and Learning, Linköping University, 58183 Linköping, Sweden; ^3^The Swedish Institute for Disability Research, Örebro University, 702 81 Örebro, Sweden; ^4^Eriksholm Research Centre, Oticon A/S, 20 Rørtangvej, 3070 Snekkersten, Denmark

## Abstract

The main focus of this paper is to discuss the importance of “evaluating the process of change” (i.e., process evaluation) in people with disability by studying their lived experiences. Detailed discussion is made about “why and how to investigate the process of change in people with disability?” and some specific examples are provided from studies on patient journey of persons with hearing impairment (PHI) and their communication partners (CPs). In addition, methodological aspects in process evaluation are discussed in relation to various metatheoretical perspectives. The discussion has been supplemented with relevant literature. The healthcare practice and disability research in general are dominated by the use of outcome measures. Even though the values of outcome measures are not questioned, there seems to be a little focus on understanding the process of change over time in relation to health and disability. We suggest that the process evaluation has an additional temporal dimension and has applications in both clinical practice and research in relation to health and disability.

## 1. Introduction

Disability and impairment have been defined in a number of ways, with either narrow or wider criteria. In general, wider criteria or definitions have been used when studying disability from social sciences perspective. Disability has also been studied, understood, and described by using various models both in practice and in research [1, 2], and some such models include biomedical, social, and biopsychosocial models [3–6]. In addition, disability has also been studied from perspectives such as systems theory [7, 8], intersectionality, and juridification [9].

Furthermore, various metatheoretical perspectives (i.e., philosophical standpoint) have been applied to disability research, for example, external realism (naïve realism), antirealism, and critical realism [10]. These metatheoretical perspectives can be complementary but some can be contradictory. Some of the perspectives are less inclusive (e.g., naïve realist and antirealist) and others are of inclusive nature (e.g., critical realism) in studying and understanding disability. In addition, the experiences of people with disability and how it may change over time can also be studied (i.e., process evaluation).

In general, the healthcare practice and work with disability management are dominated by the use of “outcome measures.” In a recent study focused on developing ICF core sets for hearing loss, it was found that there are over 100 different outcome measures in the literature related to adults with hearing impairment [11]. It was also highlighted that there are very few longitudinal studies in relation to adults with hearing impairment. Whilst the values of outcome measures are not at question, there seems to be a little focus on understanding the process of change over time in relation to health and disability. A recent study by Laplante-Lévesque et al. provides an example of study with a focus on process of change over time in adults with hearing impairment seeking help for the first time [12]. In addition, in recent years few researchers have highlighted the need for process evaluation in relation to evaluating health interventions [13, 14].

This paper aims at discussing the importance of “evaluating the process of change” (i.e., process evaluation) in understanding disability by studying their lived experiences. The paper written in two folds, in Section 2, we start with distinguishing between “outcome measurement” and “process evaluation,” and we discuss the importance of process evaluation and provide some examples from our research on hearing impairment and make discussion about why and how to investigate the process of change in a person with disability. It is important to note that whilst the discussions are generally made about disability the empirical examples are provided from studies on hearing impairment. You can refer to a paper by Manchaiah and Stephens for detailed information about terminologies and definitions about hearing impairment [15]. In Section 3, discussions are made to highlight the methodological aspects in process evaluation (i.e., how different theoretical positioning may influence the “process evaluation”) by relating it to ideas from various metatheoretical perspectives. Overall, the emphasis in Section 2 is method and the emphasis in Section 3 is to relate method to metatheory.

## 2. Outcome Measurement versus Process Evaluation

It is quite common to study and evaluate change when it comes to health and also health interventions. Outcome measures are tools used in assessing the change over time. However, in healthcare practice they are mainly used as baseline measurement during the initial consultation of the patient and after the intervention. The change in the outcome measures is usually assumed to be due to treatment and/or interventions. This is the typical design used in research trials with classical OXO model (one-group-pre-post) as proposed by Campbell and Stanley [16]. Outcome measures can have various purposes, for example, (a) to measure rehabilitative outcomes of an individual person with disability; (b) to access the effectiveness of the service provided by a particular clinical unit or agency; (c) to access the effectiveness of new technologies and treatment methods; and (d) to assess the effectiveness of rehabilitation services on quality of life [17]. In addition, outcome measures have also been used in formulating intervention strategies [18]. Whilst the outcome measures can be used longitudinally to measure change over time, they are mainly used just before and after the treatment. Furthermore, there is “*almost no research on the rate of change in outcome measures throughout the episodes of treatment or how much treatment is required to produce a valued outcome*” [19].

In recent years, there is more emphasis to measuring health outcomes in terms of function. For example, World Health Organisation-International Classification of Functioning, Disability and Health (WHO-ICF) is based on the biopsychosocial model which assumes an interplay of factors at different levels (e.g., biological, psychological, and social) and advocates understanding disability in terms of impairment, ability, activity limitations, and participation restrictions [20]. In practice, the impairment is usually measured using clinical evaluations (i.e., objective measures) and disability (i.e., activity limitations and participation restrictions) is measured using self-reported outcome measures (i.e., subjective measures).

The term “process evaluation” in this paper has been used in the context of understanding and monitoring the change longitudinally (e.g., several days to several years). This aspect relates mainly to how the experiences of people with disability or a particular health condition change over time. For example, studies on “patient journey” have become popular in recent years which evaluate main phases they go through during their disease and treatment regime. There are examples of such studies in relation to hearing impairment [21–24].

### 2.1. Importance of Process Evaluation

Whilst the uses of outcome measures are most common, it can be argued that both outcome measurement and process evaluation are important. Some inspiration for process evaluation can be drawn from the area of marketing and business studies. For example, the concept of “product life cycle” refers to the stages through which product or its category bypasses, which may include stages such as introduction to the market, growth, maturity, and decline [25, 26]. This model provides important information about the product in the temporal dimension. However, it is important to note that length of each cycle in each product varies greatly. Despite the shortcomings that it may be difficult to identify where the product is in its life cycle and almost impossible to know with certainty when a product moves from one stage to another stage, the model is still very popular in the area of marketing and business management in formulating strategy.

To better understand the difference between process evaluation and outcome measurement, let us consider a simple scenario where a person is travelling from place A to place B. His main goal in this context is to reach B. In this example, if you use only outcome measurements we can capture whether or not a person reached place B. It can include more variables, for example, within a given time limit and using a particular route, and so forth. However, it does not capture the experience through this journey and how they changed over time. More importantly, what sort of factors may have positively or negatively influenced the journey? Even though most people may consider reaching to place B as a success, some may decide not to undertake that journey again because of the difficult experiences they had through this journey or the opposite. This example may suggest that the process evaluation may to some extent highlight various factors that may not be understood through outcome measures. We can apply this way of thinking to a particular health condition and disability.

Furthermore, it is important to note that there are benefits and shortcomings of both outcome measurement and process evaluation. A discussion paper by Mant compares process and outcome measures as performance indicators in healthcare and suggested that health care is one determinant of health and there could be other factors (e.g., nutrition, environment, lifestyle, etc.), which may influence the health outcomes [27]. The differences in outcomes (which are measured using outcome measures and can reflect wide range of aspects) may be due to various reasons such as types of cases on which the treatment was administered, how the data was collected, chance, and quality of care given. However, process evaluation could have some advantages as they are more sensitive to difference in quality of care and may act as a direct measure of quality. Mant also argued that the outcome measures are of use only where outcome indicator has the power to detect variation in healthcare leading to changes in health outcome (and such changes are sufficiently common so that it will produce enough power in outcome measures) [27]. For this reason, if these conditions are not met then other approaches such as process measurement and risk management of individual incidents could be more effective rather than looking at statistical variation of data from larger samples. Even though the perspectives expressed in this paper are based on looking at healthcare practice as a whole and to discuss the strengths and weakness of outcome measures and process measures as performance indicator for healthcare, the findings and recommendations have some use to each individual.

### 2.2. Outcome Measurement

The outcome measures gives information about a specific aspect (e.g., depression, anxiety, level of hearing disability, etc.) depending on the measures we are using and also the extent to which the person is affected at that point of time. The Hearing Handicap Questionnaire (HHQ), which is used to measure the psychosocial aspects of hearing disability, is a good example of an outcome measure [28, 29]. Changes in outcome measures can be used to evaluate the degree of success of an intervention. For example, with HHQ lower perceived hearing disability after audiological management (e.g., hearing aids) suggests the benefits of the management approach used.

### 2.3. Process Evaluation

The process evaluation refers to studying the experiences of person with disability in the form of a timeline to understand the main phases/stages they go through during the disease and the treatment. Studies on patient journey represent good examples of process evaluation [21–24]. Even though studies on patient's journey uncover important information about the process of change in a person with disability and factors influencing them, they may not capture the intensity by which the person is affected at one point of time. Although there is some amount of theme identification in this, time is an additional dimension in this approach. In addition, it can be argued that the use of outcome measures at multiple intervals may act as process evaluation (i.e., continuous monitoring of outcomes using outcome measures). However, devising such a measurement tool to capture both outcome and process could be challenging.

Overall, it is important to note that even though the approaches discussed above provide similar information, they give different perspectives in understanding the same condition. It can be argued that the combination of such approaches may give better understanding of a person with disability than any other approach alone. It is also important to establish a link between such combined approaches in order to better understand what information they are providing. This discussion may highlight the fact that process evaluation has some use in disability research and may also have some clinical value.

### 2.4. Process Evaluation: Examples from Studies on Hearing Impairment

It appears in the recent years that studying the lived experiences of persons with disability is becoming popular. Moreover, there is an “*increase in the instances that voices of disabled people are being heard or considered in all stages of research about their lives*” [1, 30]. Studies on patient journey represent one way of capturing the lived experiences of people with disability. In addition, such studies also explore the process of change by considering various experiences a person may have during the initial onset of the disease and realising that they have the condition, acceptance and help-seeking, assessment, rehabilitation, and continued experience living with a particular disability. Reported experiences can be analysed to identify relevant themes and be represented in the way the themes reflect the data. Such an approach is often used in disability research while attempting to understand the lived experiences of a person. A recent international study on perspectives of adults with hearing impairment towards help-seeking and rehabilitation is a good example of such an approach [31]. In a clinical setting this is done informally through case history.

**Figure 1:**
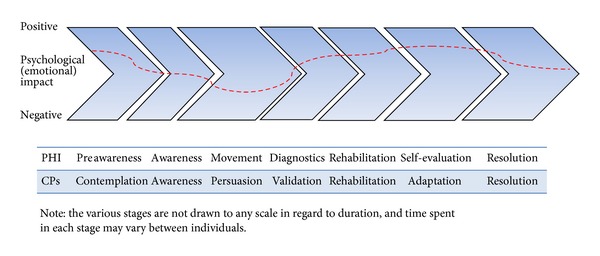
Models of persons with hearing impairment (PHI) and their communication partner's (CPs) journey through hearing impairment.

The Ida Institute initially developed the possible patient journey of person with hearing impairment (PHI) and their communication partners (CPs) by considering the professionals' perspective [32, 33]. Our studies further developed these models by considering perspectives of PHIs and their CPs [21–24]. These models are indicated in Figure 1 which demonstrates the main phases the PHI and their CPs go through from the initial onset of the disease, diagnosis, treatment, and then continuing to live with the hearing impairment. It is important to note that the various stages in the model are not drawn to any scale and the progression from one phase to other may vary from person to person quite considerably (i.e., several days to several years). Even though the above model demonstrates the process of change from the perspective of PHI and their CPs, it does not measure the intensity at which they are affected at any one stage/point. For this reason, outcome measures would be helpful in measuring the intensity at which the person is affected at any point of time.

Studies on patient journey of PHI and CPs look at hearing disability from a different dimension (i.e., temporal) and have given some new insights. Moreover, studies on patient journey can also highlight some of the barriers to help-seeking process which may not be identified through structured outcome measurements. For example, in our recent study on sudden-onset hearing loss patient journey patients reported that the medical professionals did not always give them the correct information about the condition and expected prognosis which raises some general, ethical, and legal issues [23, 34].

The phases represented in these patient journey studies related to hearing impairment seem to correlate well with the stages of change proposed in “transtheoretical model of change” (also known as stages of change theory) which was proposed in relation to health behaviour change [35, 36]. This theory suggested that health behaviour change involves six main stages, which include precontemplation, contemplation, preparation, action, maintenance, and termination. This model is cyclic or spiral model rather than linear which accounts for relapse and a restart. Such a model could be helpful while understanding the process of change through a disease and its treatment. The WHO has recently produced a document about “engaging in process of change,” which highlights some facts and methods used to engage in the process of change [37].

### 2.5. How to Best Evaluate the Process of Change in a Person with Disability?

Process evaluation can be studied and understood from perspectives of person with disability, their significant others (e.g., spouse, children, relatives, friends), and clinicians and in the wider context of society. However, it is important to note that priorities from each of these perspectives could be different. For example, (a) for people with disability and their significant others, their activity, participation, and quality of life could be key factors; (b) for clinicians, cure of impairment, reducing disability, and to some extent quick fix to the problems reported could be important; and (c) for society, less dependency of people with disability on society and a larger contribution from them could be important. Even though it is difficult and to some extent impossible to answer which of these perspectives are more important, considering the emphasis on “shared decision making” in recent years, the combined approach could be helpful. Moreover, the process of change can also be evaluated from different analytical levels, which may include biological, psychological, psychosocial, and socioeconomic.

In our research on patient journey of PHI and their CPs, we have focused on individual level by studying the reports of the PHI, their CPs, and clinicians [21–24]. Moreover, these studies had relatively small sample sizes and employed research designs, which were based on reported experiences and may have been influenced by various aspects including perceptions and memory of the individuals. However, considering that the journey of PHI and their CPs may take several years, longitudinal designs may be more appropriate. In addition, evaluating such models in a large population is necessary.

## 3. Metatheoretical Approaches and Their Implications to Process Evaluation

As discussed in the earlier section disability and impairment have been studied and understood using various metatheoretical perspectives. In this section we will consider three perspectives and discuss their advantages and limitations. Detailed information about the metatheoretical perspectives in relation to the assessment of audiological rehabilitation can be found in the paper by Danermark [10].

Metatheory refers to a formal system that describes the structure of some other system (i.e., a theory about theory). Metatheory belongs to the philosophical specialty of epistemology (i.e., knowledge production) and also to assumptions about reality (ontological issues). The metatheoretical approaches are the key aspects, which determine our views on reality, to what extent it is possible to gain in-depth knowledge about reality, and also how we choose to study a particular phenomenon and/or problem. There are various metatheoretical approaches which can be grouped in some main categories: naïve realism (or empiricism), antirealism (e.g., social constructionism), critical realism, neo-Kantianism, and hermeneutics [10, 38–40].

It is important to note that all these perspectives have benefits and shortcomings and some of them are more inclusive when it comes to disability research than others. Moreover, they have some key assumptions. For example, (a) naïve realism is the most common approach which assumes that reality can be studied and understood by collecting empirical evidence in an objective nature and our senses help us in understanding reality (i.e., our senses provide us with direct knowledge of the external world); (b) antirealism (e.g., social constructionism) is that reality is socially constructed and there is no reality independent of our perception (or there is no objective truth and we can only understand the reality by personal accounts of people experiences which could differ) [41, 42]; and (c) critical realism is that reality exists at different analytical levels (i.e., stratified) and there is reality independent of our knowledge about it (or we can get closer to reality by understanding different levels of analytical truth and its interaction, but we may never get the complete truth). Moreover, critical realists make a distinction between empirical (i.e., our experience of what actually happens); actual (i.e., all the things that happen independently whether they are observable or not); and real (i.e., reality consists of mechanisms with generative powers).

Even though any of these three metatheoretical approaches can be used in studying hearing impairment from each of the methods discussed above (i.e., outcome measurement, process evaluation, and identifying themes from narratives), some approaches have been used for a certain dimension more often than others. For example, outcome measurements, which provide numerical quantification, have been an approach from a naïve realist perspective; and identifying themes from narratives has been the choice for antirealists.

Critical realism has a great advantage in process evaluation but also in the other two dimensions when compared with naïve realism and antirealism. This is because the “process evaluation” involves similar aspects which could be studied using outcome measures or identifying themes through reported experiences; however, this requires recontextualization and redescription of data from different theoretical perspective. For example, in patient journey studies the stages of change theory have been used to study the process of change through PHI and their CPs journey through hearing loss.

Moreover, one of the important steps in research from critical realist perspective is “abduction inference” which refers to “*interpret and recontextualise individual phenomenon within a conceptual framework or a set of ideas, to be able to understand something in a new way by observing and interpreting this something in a new conceptual framework*” [43]. There are no fixed criteria from which the abduction inference is done. However, this involves recontextualization and redescription of data, creativity, and imagination. It is important to note that most researchers follow such a process even though they may not be aware of the term abduction inference. This is an important concept in the process evaluation (i.e., studying process of change). This is because the experiences reported in the PHI and their CP journey studies can be found in the previous literature, for example, in a recent international study on perspectives of adults with hearing impairment towards help-seeking and rehabilitation [31]. However, the patient journey studies involved new theoretical framework (i.e., stages of change theory), which evaluates the experiences of hearing impairment and how they change over time.

Critical realism is also a fruitful perspective when it comes to outcome measures, because this perspective clearly demonstrates the boundaries for drawing conclusions based on quantitative analysis in order to avoid conclusions about causality that is beyond the capacity of the conducted research. It highlights the importance to not only include the “surface” in terms of observed empirical events but also include analysis of underlying structures and mechanisms [43]. Furthermore it also stresses the importance to take the contexts into consideration (i.e., mechanisms + context = outcome). In short it aims for answering the question “*for whom, works what in which context?*.” However, ultimately the aspiration is to make the results generalisable.

## 4. Discussion

Disability is a complex phenomenon, which needs to be studied and managed with a holistic perspective. Moreover, disability experienced by an individual due to a specific condition (e.g., hearing impairment) may be diverse in its nature. Kerr and Cowie suggested that “*impact of acquired deafness cannot be understood simply by measuring its intensity and documenting the objective limitations that it imposes*” [44]. For this reason, it is important to clarify and understand why acquired hearing disability affects people the way it does [45]. However, this may require a multidimensional approach and it appears that studying “lived experiences” could be important.

Söder argued that there are various tensions in disability research (e.g., theory and political action, impairment versus disability, and theoretical and empirical research) [9]. Such tensions could arise from the metatheoretical approaches we take. For example, naïve realists believe that our sense provides us with the direct knowledge of the external world. According to this perspective empirical observations are central to research and objective assessment is the key aspect of gaining knowledge. Naïve realism approach to process evaluation requires quantification. This way of precisely quantifying which stage the PHI might be at one point could be done by designing structured questionnaires to capture different phases of the journey.

However, according to antirealists no objective statements about reality are possible due to the distinction first formulated by the German philosopher Emanuel Kant, things-in-themselves versus things-for-us (German:* Dinge-an-sich versus Dinge-für-uns*), and hence negotiation is important to come to agreement of what is working and what is not working in, for example, audiological rehabilitation. A quotation “*there are no facts, only interpretations*” is a good example of this way of thinking [46]. This statement illustrates that everything is subject for negotiations and there is no right or wrong based on objective facts. If we take antirealists (e.g., social constructionists) view it may not be possible to come up with any model, which can be empirically generalised to large group of population as it is against their underplaying assumption towards reality.

From a critical realist perspective to process evaluation, it is important to have theories about how mechanisms are working in different contexts and how they produce the outcomes. Specifically, when such a concept is applied to patient journey studies, it is important to consider forming theories on how patient journey may be influenced by various biological, social, cultural, and economical aspects. Such an approach is challenging and requires interdisciplinary work. Moreover, critical realism as a metatheoretical choice may have advantages to process evaluation, as the concepts such as “abduction inference” are central to research from critical realist perspective.

Overall, it is important to note that disability and impairment have been be studied and understood from various models and perspectives and there are significant differences in how it is understood based on the metatheoretical perspectives we take. The discussion made in this paper and also our studies on patient journey highlight the importance of process evaluation based on critical realism and suggest that such an approach could make an important contribution to better understand disability. We suggest that the process evaluation has an additional temporal dimension and has applications in both clinical practice and research. Furthermore, whether to choose the metatheoretical approaches based on the aim (e.g., outcome measurement or process evaluation) or vice versa (i.e., metatheoretical positioning guiding the choice of method) may create a dilemma for researchers. However, it is most important that the researchers are aware of the benefits and shortcomings of the choices made and also that such aspects should be discussed while reporting the research findings.
